# Metabolic and renal changes in patients with chronic hepatitis C infection after hepatitis C virus clearance by direct‐acting antivirals

**DOI:** 10.1002/jgh3.12324

**Published:** 2020-03-20

**Authors:** Riccardo Nevola, Luca Rinaldi, Letizia Zeni, Ferdinando C Sasso, Pia C Pafundi, Barbara Guerrera, Aldo Marrone, Mauro Giordano, Luigi E Adinolfi

**Affiliations:** ^1^ Department of Advanced Medical and Surgical Sciences University of Campania “Luigi Vanvitelli” Naples Italy; ^2^ Department of Translational Medical Sciences University of Campania “Luigi Vanvitelli” Naples Italy; ^3^ Clinical Hospital of Marcianise, ASL Caserta Caserta Italy

**Keywords:** direct‐acting antivirals, estimated glomerular filtration rate, insulin resistance, low‐density lipoprotein cholesterol, serum cholesterol, serum glucose

## Abstract

**Background and Aim:**

The impact of hepatitis C virus (HCV) clearance by direct‐acting antiviral agents (DAAs) on HCV‐related extrahepatic manifestations is not well known. We evaluated the effect of viral clearance on metabolic and renal parameters.

**Methods:**

In this prospective study, HCV patients who achieved a sustained virologic response (SVR) by DAAs were evaluated before, at the end, and 24 weeks after treatment for glycemic (serum glucose and insulin, HOMA‐IR, HOMA‐β, and HOMA‐S) and lipid (serum cholesterol, triglycerides, low‐density lipoprotein [LDL], high‐density lipoprotein) metabolism and renal function (serum creatinine, estimated glomerular filtration rate [eGFR]).

**Results:**

A total of 343 consecutive HCV patients were evaluated. At 24 weeks of post‐follow‐up, an increase in body mass index (BMI) was observed (*P* < 0.05). Regardless of hepatic fibrosis levels and BMI, a reduction in serum glucose (*P* = 0.001), HOMA‐IR (*P* < 0.001) and HOMA‐β (*P* < 0.001) and an increase in HOMA‐S (*P* < 0.001) values were observed at 24 weeks after HCV clearance as compared to pretreatment values; 32.4% of patients with impaired fasting glucose normalized serum glucose values and 44.6% of diabetics showed an improvement in glycemic control. In contrast, serum cholesterol (*P* < 0.001) and LDL cholesterol (*P* < 0.001) values were increased. Renal function was improved with about 10% reduction of serum creatinine values (*P* < 0.02) and an increase of eGFR (*P* < 0.001). A baseline eGFR of ≤60 mL/min/1.73 m^2^ was a negative predictor of renal function improvement. HCV clearance was an independent factor improving glucose metabolism and renal function.

**Conclusions:**

Our study shows an occurrence of changes in metabolic and renal parameters in HCV patients with SVR, anticipating possible future clinical scenarios that the clinician must know for proper management.

## Graphical abstract

In a prospective study, 343 patients with hepatitis C virus (HCV) infection who achieved sustained virologic response by direct‐acting antiviral agent (DAA) treatments were followed up for 24 weeks to evaluate the impact of viral clearance on metabolic and renal parameters. HCV clearance was independently associated with a significant reduction in serum glucose, HOMA‐IR, and HOMA‐β and an increase in HOMA‐S. Furthermore, an increase in serum cholesterol and low‐density lipoprotein cholesterol levels was observed. Renal function was improved with a significant decrease in serum creatinine levels and an increase in estimated glomerular filtration rate. Data show that HCV clearance by DAA induces significant changes in metabolic and renal parameters.

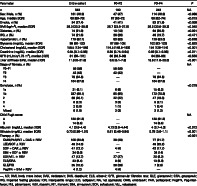

## Introduction

Chronic hepatitis C virus (HCV) infection is currently one of leading causes of liver disease, affecting 71.1 million of patients worldwide.^1^ Beyond the effect of HCV infection on the liver, the virus induces several systemic manifestations.^2^ The HCV‐associated extrahepatic manifestations have an impact on morbidity and mortality in these patients. HCV is closely related to disorders of glucose metabolism, such as insulin resistance (IR) and type 2 diabetes mellitus (T2DM). IR is associated with HCV infection in up to 80% of cases and the risk of developing T2DM is twice higher than that of subjects without HCV.^3^ Moreover, a reduction in serum levels of total cholesterol, low‐density lipoprotein (LDL), and very‐low‐density lipoprotein (VLDL) occurred, and these findings are related to the role played by lipids in the HCV life cycle.^4^

HCV‐infected patients showed also a higher risk of developing both chronic kidney disease (CKD) and a faster progression toward end‐stage renal disease (ESRD) compared with patients with no HCV infection.^5^, ^6^

The recent introduction of direct‐acting antiviral agents (DAAs) has revolutionized the therapeutic approach. A sustained virologic response (SVR) is achieved in up to 99% of patients treated with DAAs. Considering the direct or indirect role of HCV on metabolic and renal changes, it is hypothesized that the clearance of HCV by DAA regimens may modify metabolic and renal parameters in these patients and that knowledge of such changes is essential for planning best follow‐up.

Consequently, the aim of this study was to evaluate whether in chronic hepatitis C patients the viral clearance by DAAs influences the metabolic and renal function.

## Methods

### *Study design*

In the current prospective study, from May 2016 to May 2018, all patients with chronic HCV infection referred to our hepatologic center who underwent DAA treatments were enrolled. Exclusion criteria were DAAs nonresponder, relapse, coinfection with hepatitis B virus or human immunodeficiency virus, and history of alcohol use (>3 drinks per day).

### *Assessments*

Patients were evaluated before starting treatment, at the end of treatment, and 24 weeks after treatment discontinuation. Liver and kidney function tests, fasting blood glucose and insulin levels, blood counts, serum cholesterol, LDL and high‐density lipoprotein (HDL) cholesterol, and triglycerides levels were evaluated in all patients before, at the end and 24 weeks after treatment suspension.

The diagnosis of T2DM and impaired fasting glucose (IFG) was done according to American Diabetes Association's guidelines.^7^ Serum creatinine levels were assessed by the high‐performance liquid chromatography (HPLC) method with a reference range of 0.67 to 1.17 mg/dL. Estimated glomerular filtration rate (eGFR) was calculated by the MDRD formula (in its six‐variable form).^8^ HCV‐RNA levels were assessed by real‐time polymerase chain reaction and the viral genotype was identified by Innolipa assay. Serum insulin levels were assessed by immunoassay for human insulin (Insulin Cobas, Roche Diagnostics, Indianapolis, IN, USA).

HOMA was used to evaluate IR and β‐cell function by the following formulas: HOMA‐IR = ([glycemia (mg/dL) × insulinemia (μU/mL)]/405); HOMA‐β% = (360 × fasting insulin μU/mL)/(fasting glucose mg/dL‐63); and HOMA‐S% = 1/(log(fasting insulin μU/mL) + log(fasting glucose mg/dL). The cutoff for the interpretation of HOMA‐IR data was obtained on a population of 130 healthy Caucasian subjects from the same geographical area and was equal to 2.60 (75th percentile of HOMA‐IR).^9^, ^10^

Prior to the initiation of antiviral treatment, patients were evaluated for body mass index (BMI) (kg/m^2^) and underwent abdominal ultrasound and transient elastography (TE) using Fibroscan (Echosens, Paris, France) to assess the degree of liver fibrosis.

Antiviral treatment was performed using DAAs according to the EASL guidelines.^11^

### *Statistical analysis*

Data are shown as median and interquartile range, in the case of continuous variables and number and percentage, for categorical variables. The normal/not normal distribution was preliminarily assessed through a Kolmogorov–Smirnov (K‐S) goodness‐of‐fit test. Continuous variables were assessed, in the case of normal distribution, by either a paired *t* test for two sets of observations or repeated measures anova for more than two longitudinal observations. Where variables were not normally distributed, appropriate nonparametric tests were used. In particular, the Wilcoxon signed rank test and Friedman test, depending on the number of repeated observations, were used. Comparisons along time between the two groups were evaluated by mixed model design. A Cox regression model was used to assess predictive factors for HOMA‐IR and eGFR improvement after adjusting for HOMA‐IR and eGFR at baseline. The data are expressed as hazard ratio (HR) with 95% confidence interval (CI) for the univariate analysis and as adjusted HR (aHR) (95% CI) for the multivariate analysis. *P* values below 0.05 were considered statistically significant. All analyses were performed with the SPSS 24 software (IBM, Armonk, NY, USA).

## Results

Three hundred forty‐nine patients with chronic HCV infection were treated with DAAs during a 2‐year period and evaluated for this study. Of these, 343 patients (98.3%) who achieved SVR were closely followed up for 24 weeks after the end of treatment, and data are reported in the present study. The characteristics of the study population are shown in Table 1.

**Table 1:** Baseline characteristics of the study population

Parameter	Entire cohort	F0–F2	F3–F4	*P*
*n*	343	100	243	NA
Sex: Male, *n* (%)	161 (46.9)	47 (47)	114 (46.9)	<0.988
Age, median [IQR]	68 [59–73]	67 [53–72]	68 [62–74]	<0.013
Smoke, *n* (%)	94 (27.4)	41 (41)	53 (21.8)	<0.008
BMI (kg/m^2^), median [IQR]	26.0 [23.2–28.6]	25.7 [22.9–27.9]	26.2 [23.6–29.2]	<0.040
Diabetes, *n* (%)	74 (21.6)	10 (10)	64 (25.3)	<0.001
IFG, *n* (%)	74 (21.6)	26 (26)	48 (19.7)	<0.471
Hypertension, *n* (%)	179 (52.1)	50 (50)	129 (53.0)	<0.626
Glycemia (mg/dL), median [IQR]	99 [90–120]	97 [88–108]	102 [93–123]	<0.533
Cholesterol (mg/dL), median [IQR]	159.5 [124–195]	174.5 [149.5–193]	154 [126–174]	<0.001
Creatinine (mg/dL), median [IQR]	0.84 [0.7–0.98]	0.85 [0.75–0.94]	0.83 [0.7–0.96]	<0.071
GFR (mL/min/1.73 m^2^), median [IQR]	81 [68–101]	80 [68.5–98]	84 [68–99]	<0.535
Liver stiffness (kPa), median [IQR]	11.8 [8–21.8]	6.5 [5.1–7.8]	16.5 [11.6–26.3]	<0.001
Stage of fibrosis, *n* (%)				NA
F0–F1	58 (58)	58 (58)	—	
F2	42 (42)	42 (42)	—	
F3	79 (32.5)	—	79 (32.5)	
F4	164 (67.5)	—	164 (67.5)	
Genotype, *n* (%)				<0.279
1a	21 (6.1)	6 (6)	15 (6.2)	
1b	237 (69.1)	65 (65)	172 (70.8)	
2	69 (20.1)	22 (22)	47 (19.3)	
3	8 (2.3)	3 (3)	5 (2.1)	
4	2 (0.6)	1 (1)	1 (0.4)	
Mixed	6 (1.8)	3 (3)	3 (1.2)	
Child Pugh score				NA
A	150 (91.5)	—	150 (91.5)	
B	14 (8.5)	—	14 (8.5)	
Albumin (mg/dL), median [IQR]	4.1 [3.8–4.4]	4.13 [3.8–4.44]	4 [3.7–4.3]	<0.012
Bilirubin (mg/dL), median [IQR]	0.73 [0.56–1.01]	0.61 [0.46–0.84]	0.78 [0.6–1.1]	<0.001
Therapy, *n* (%)				<0.001
OMB/PAR/RIT + DAS ± RBV	105 (30.6)	28 (28)	77 (31.7)	
LED/SOF ± RBV	49 (14.3)	—	49 (20.2)	
SOF + DAK ± RBV	47 (13.7)	4 (4)	43 (17.7)	
SIM + SOF ± RBV	34 (9.9)	—	34 (14)	
SOF/VEL ± RBV	47 (13.7)	27 (27)	20 (8.2)	
ELB/GRA	22 (6.4)	9 (9)	13 (5.3)	
GLE/PIB	35 (10.2)	28 (28)	7 (2.9)	
PegIFN + SIM + RBV	4 (1.2)	4 (4)	—	

—, ND; BMI, body mass index; DAK, daclatasvir; DAS, dasabuvir; ELB, elbasvir; GFR, glomerular filtration rate; GLE, glecaprevir; GRA, grazoprevir; IFG, impaired fasting glucose; IQR, interquartile range; LED, ledipasvir; NA, not applicable; OMB, ombitasvir; PAR, paritaprevir; PegIFN, Peg‐interferon; PIB, pibrentasvir; RBV, ribavirin; RIT, ritonavir; SIM, simeprevir; SOF, sofosbuvir; VEL, velpatasvir.

To discern the effect of liver fibrosis levels on results, the study population was divided in two subgroups according to the degree of liver fibrosis: group one included subjects with absence to moderate hepatic fibrosis (F0–F2; *n* = 100) and group two with advanced hepatic fibrosis or liver cirrhosis (F3–F4; *n* = 243). The cu‐off used to distinguish the two groups was 10 kPa of liver stiffness. Patients with liver cirrhosis (*n* = 164) were mostly classified as Child Pugh class A scores (91.5%). The most frequent genotype was genotype 1b (237 patients, 69.1%), followed by genotype 2 (69 patients, 20.1%). The DAA regimens used are shown in Table 1; the duration of treatments ranged from 8 to 24 weeks.

As shown in Table 1, the average age was 68 years, and 47% of patients were male. The median baseline BMI was 25.8 and steatosis was present in 32% of cases. T2DM was present in 74 (21.6%) patients, and IFG was observed in 74 (21.6%) patients. A serum cholesterol greater than 200 mg/dL was observed in 33 patients, and 13 (3.7%) were on lipid‐lowering agents (statins). In addition, patients with advanced hepatic fibrosis or liver cirrhosis (F3–F4) were significantly more frequently affected by diabetes mellitus (25.3 *vs* 10%, *P* < 0.001). Conversely, total cholesterol levels were significantly lower in the F3–F4 group (median 174.5 mg/dL *vs* 154 mg/dL; *P* < 0.001).

### *Impact of HCV eradication by DAAs on the anthropometric, glycemic, and lipid profile*

Table 2 shows changes in metabolic profile and anthropometric data in the above‐mentioned groups, once the HCV was cleared by DAAs. After HCV clearance and at the end of follow‐up, a significant increase in BMI was observed in both groups (Table 2). To avoid bias, no concomitant changes were performed in antihypertensive, hypolipidemic, or hypoglycemic therapy during the study observation period. For this purpose, any kind of alcohol intake was discouraged. In the end, no objective variation of the patients' life habits (e.g. diet, exercise) was found.

**Table 2:** Anthropometric, metabolic, and renal profile before and after hepatitis C virus clearance

Parameters	F0–2	*n*	*P*	F3–4	*n*	*P*	*P* †
Fasting glucose (mg/dL), median [IQR]		100	<0.027		243	<0.001	<0.001
T0	97 [88–108]			102 [93–123]			
EOT	94 [86–107]			99 [90–112]			
SVR24	91 [84–102]			94 [88–110]			
Fasting insulin (μU/mL), median [IQR]		90‡	<0.001		179‡	<0.001	<0.027
T0	12 [8–16]			16 [10.85–22]			
EOT	10.0 [6.5–13.8]			12.5 [9–17.8]			
SVR24	9.1 [5.2–11]			10.5 [7.3–14]			
HOMA‐IR, median [IQR]		90‡	<0.001		179‡	<0.001	<0.001
T0	2.85 [2.11–4.07]			4.4 [3.4–5.3]			
EOT	2.52 [1.69–3.45]			2.8 [1.8–4.1]			
SVR24	2.15 [1.63–2.6]			2.5 [2–3]			
Total cholesterol (mg/dL), median [IQR]		100	<0.001		243	<0.010	<0.457
T0	174 [149.5–193]			154 [126–174]			
EOT	190 [172.2–218.9]			173 [148.5–203.5]			
SVR24	192 [172.3–218]			169 [134–190]			
LDL cholesterol (mg/dL), median [IQR]		100	<0.001		243	<0.011	<0.008
T0	99 [83.8–125.5]			89 [65–107]			
EOT	119 [103.5–14 352]			97 [77–125]			
SVR24	118 [97–133.5]			103 [87.7–121.3]			
HDL cholesterol (mg/dL), median [IQR]		100	<0.001		243	<0.476	<0.370
T0	50 [39–57.7]			50 [40–59.5]			
EOT	50 [40–60.3]			50 [40–59.8]			
SVR24	49 [42–60]			48 [37–57]			
Triglycerides (mg/dL), median [IQR]		100	<0.001		243	<0.731	<0.001
T0	93.5 [70.8–115]			93 [76.3–122.5]			
EOT	106 [77–139]			92 [71–131]			
SVR24	102 [80–134]			92 [68.3–122.8]			
BMI (kg/m^2^), median [IQR]		100	<0.005		243	<0.048	<0.089
T0	25.7 [22.9–27.9]			26.2 [23.6–29.2]			
EOT	26.3 [23.1–28.2]			26.8 [23.5–30.1]			
SVR24	26.6 [23.1–28.6]			27.1 [24.1–30.3]			
Creatinine (mg/dL), median [IQR]		100	<0.001		243	<0.001	<0.377
T0	0.85 [0.75–0.94]			0.83 [0.70–0.96]			
EOT	0.82 [0.71–0.91]			0.82 [0.67–0.90]			
SVR24	0.78 [0.71–0.89]			0.76 [0.66–0.90]			
Glomerular filtration rate (mL/min/1.73 m^2^), median [IQR]		100	<0.001		243	<0.001	<0.066
T0	80 [68.5–98]			84 [68–99]			
EOT	82 [70–108]			83 [66–98.6]			
SVR24	90 [76–109]			92 [73.5–108]			

† Mixed model analysis assessing differences along times between groups.

‡ Diabetics were excluded.

BMI, body mass index; EOT, end of treatment; HDL, high‐density lipoprotein; IQR, interquartile range; LDL, low‐density lipoprotein; SVR24, sustained virological response at 24 weeks posttreatment.

In addition, in a control group of 104 patients with HCV (98 awaiting treatment with HCV plus 6 patients without response from this study), serum fasting glucose, cholesterol, and triglycerides levels were assessed during a 4‐month period. There were no significant changes in the values of fasting blood glucose (baseline: 98 mg/dL [91–119] and 4 months: 97.8 mg/dL [90–118]), serum cholesterol levels (baseline: 158 mg/dL [128–192] and 4 months: 156 mg/dL [126–194]), and triglycerides levels (baseline: 94 mg/dL [72–114] and 4 months: 93 mg/dL [74–116]). These data agree with those already published by our group in 65 HCV untreated patients evaluated during the same time period.^12^

#### *Changes in glycemic metabolism*

As shown in Table 2, viral eradication was associated with a significant reduction in blood glucose levels and fasting insulin in both groups (F0–F2 and F3–F4) at the end of treatment. The significant reduction, even more remarkable, was maintained after 24 weeks of posttreatment follow‐up. The decline in serum insulin levels varied accordingly to changes in blood glucose, at the end of treatment and of follow‐up (Table 2). Thus, a significant decline in HOMA‐IR values together with an improvement in the IR was observed. Notably, baseline HOMA‐IR values were significantly higher in the F3–F4 fibrosis group compared with the F0–F2 fibrosis group. Moreover, 57.1% of patients with IR had a total regression of IR status, whereas 32.5% showed a significant improvement. Overall, 89.6% of patients with IR before treatment obtained a significant regression or improvement in IR after HCV clearance and this result was confirmed at the end of the follow‐up. In patients in whom IR did not change, higher levels of hepatic fibrosis was observed with respect to those had an improvement of IR (average kPa 18.1 ± 12.2 *vs* 10.2 ± 6.4, respectively; *P* < 0001).

After HCV clearance, a significant increase in HOMA‐S was observed at the end of treatment and after 24 weeks of follow‐up with respect to baseline (102 ± 64.6 and 106 ± 66.2 *vs* 78.4 ± 54.2, respectively, *P* < 0.001), whereas a significant reduction in HOMA‐β was observed (121.4 ± 46.4 and 116.2 ± 42.4 *vs* 138.6 ± 66, respectively, *P* < 0.001).

Table 3 shows the multivariate analysis of predictive factors for improvement in HOMA‐IR after adjustment for baseline values. Data show that SVR was an independent predictor of HOMA‐IR improvement (aHR, 0.266; 95% CI, 0.291–0.688; *P* < 0.008).

**Table 3:** Rates of patients who showed HOMA‐IR improvement† in in the whole treated population‡ and predictive factors for improvement

			Adjusted univariate analysis§			Adjusted multivariate analysis§		
Parameter	Improvement	No improvement	HR	95% CI	*P*	aHR	95% CI	*P*
All patients (%)	234 (67)	115 (33)	—	—	—	—	—	—
Age (years), median [IQR]	67 [58–73]	69.5 [63–74]	0.932	0.899–1.004	<0.029	0.963	0.938–0.989	<0.006
Sex: Male, *n* (%)	110 (46.6)	54 (47.8)	1.309	1.266–2.206	<0.682	1.022	0.591–1.769	<0.938
Hypertension, *n* (%)	124 (49.6)	56 (49.6)	1.069	0.794–1.712	<0.453	1.215	0.715–2.065	<0.472
Total cholesterol (mg/dL), median [IQR]	162 [133.5–182]	156 [135.5–179]	0.981	0.757–1.056	<0.486	0.998	0.990–1.016	<0.600
Cirrhosis, *n* (%)	104 (44.1)	62 (54.9)	0.896	0.911–1.743	<0.238	0.641	0.373–1.101	<0.107
SVR24, *n* (%)	236 (99.6)	107 (95.6)	0.319	0.266–0.984	<0.024	0.266	0.291–0.688	<0.008

† Improvement defined as an increase HOMA‐IR Index within reference ranges or a 50% improvement with respect to the excess from reference ranges^2^.

‡ 243 SVR patients plus 6 non‐SVR patients.

§ Adjusted for baseline HOMA‐IR.

—, NA; 95% CI, 95% confident intervals; aHR, adjusted hazard ratio; HR, hazard ratio; IQR, interquartile range; SVR24, sustained virological response at 24 weeks posttreatment.

Among the 74 HCV patients with IFG before treatment, 32.4% normalized glucose values at the end of 24 weeks follow‐up.

After elimination of HCV, 5.8% of the 74 diabetic patients had a reduction in blood glucose levels in the IFG range and their glycemic control no longer required any medication but only dietary regulation. In 38.8% of diabetic patients, an improvement in glycemic control was observed after HCV clearance, and these patients reduced the dose of antidiabetic drugs, particularly insulin by 20 to 36% of the dose used before HCV clearance.

#### *Changes in lipid metabolism*

As shown in Table 2, a significant increase in serum total cholesterol levels and a significant increase in LDL were observed after HCV eradication. To note, the increase in LDL in the F0–F2 group was more remarkable both at the end of the treatment and at the end of the follow‐up. A significant reduction in HDL levels was observed after viral clearance in the F0–F2 group only. Serum triglyceride levels showed a significant increase in the F0–F2 group after HCV clearance.

#### *Changes in renal function*

The effect of antiviral treatments with DAAs on renal function is shown in Table 2. To avoid bias, cases of acute renal failure were not included in the cohort of patients.

At the end of the follow‐up, the SVR was associated with a significant improvement in renal function in both F0–F2 and F3–F4 groups. There was a significant decrease in serum creatinine levels and a significant increase in eGFR, regardless of the degree of liver fibrosis (Table 2). Indeed, no significant differences were observed in the glomerular filtration rate improvement between the absent to mild fibrosis group and the advanced fibrosis group.

Table 4 shows the multivariate analysis of predictive factors for improvement in eGFR after adjustment for eGFR at baseline. Data show that SVR was an independent predictor of eGFR improvement (aHR, 0.058; 95% CI, 0.006–0.532; *P* < 0.012), while age was a negative predictive factor.

**Table 4:** Rates of patients who showed estimated glomerular filtration rate (eGFR) improvement† in the whole treated population‡ and predictive factors for improvement

Parameter	Improvement	No improvement	Adjusted univariate analysis§			Adjusted multivariate analysis§		
			HR	95% CI	*P*	aHR	95% CI	*P*
All patients (%)	237 (67.9)	112 (32.1)	—	—	—	—	—	—
Age (years), median [IQR]	67 [58–73]	69.5 [63–74]	0.956	0.922–0.991	<0.014	0.954	0.926–0.983	<0.002
Sex: Male, *n* (%)	110 (46.6)	54 (47.8)	1.319	0.722–2.410	<0.264	1.269	0.716–2.249	<0.414
Hypertension, *n* (%)	124 (49.6)	56 (49.6)	1.055	0.589–1.888	<0.858	1.148	0.607–2.172	<0.670
Diabetes, *n* (%)	48 (20.3)	26 (24.3)	0.784	0.389–1.580	<0.496	0.721	0.363–1.431	<0.350
Total cholesterol (mg/dL), median [IQR]	162 [133.5–182]	156 [135.5–179]	0.995	0.986–1.019	<0.291	0.997	0.988–1.006	<0.493
Cirrhosis, *n* (%)	104 (44.1)	62 (54.9)	1.443	0.786–2.647	<0.289	1.355	0.772–2.377	<0.290
SVR24, *n* (%)	236 (99.6)	107 (95.6)	0.060	0.006–0.584	<0.014	0.058	0.006–0.532	<0.012

† Improvement defined as an increase of at least 10 mL/min/m^2^.

‡ 243 SVR patients plus 6 non‐SVR patients.

§ Adjusted for baseline eGFR.

95% CI, 95% confident interval; aHR, adjusted hazard ratio; HR, hazard ratio; IQR, interquartile range; SVR24, sustained virological response at 24 weeks posttreatment.

### *Mixed model comparison over time between fibrosis stages*

A mixed model design was used to compare repeated longitudinal changes over time in between the subgroup. First, we computed generalized linear models with the best BIC and AIC, hence the most plausible ones. The linear model which best fitted to our data was that using fibrosis stage as dependent dichotomic variable (F0–F2 *vs* F3–F4), and fasting glucose; fasting insulin; HOMA index; creatinine; eGFR; total, HDL, and LDL cholesterol; triglycerides, and BMI as independent values. Fixed effects were insulin, cholesterol, renal function, BMI, and triglycerides, while we used fasting glucose and HOMA index as random effect.

As observed from the findings in Table 2, we can postulate that a higher stage of fibrosis significantly affects all glycemic parameters, while no relevant change appears in lipids control. For what concerns the renal function instead, only a trend for an increase of eGFR appears in F3–F4 subgroup. Hence, important is the use of the random effect, which significantly affects the stage of the disease.

### *Impact of HCV eradication on metabolic and renal function in the different subpopulations*

The analysis of the data considering the baseline characteristics of the various subgroups of patients has shown in Table 5. Patients with and without diabetes, normal cholesterol, with and without hypertension, older than 70 years, and with eGFR >60, after HCV clearance, showed a significant reduction in serum glucose levels, an increase in cholesterol levels, and an improvement in eGFR as observed in the analysis of the overall population. Similarly, the different therapeutic DAA regimens used with or without ribavirin did not influence the metabolic and renal changes observed in the general population (Table 5). Instead, no significant improvement in renal function was observed in subgroups of patients with hypercholesterolemia and with an eGFR ≤60 (Table 5), so these basal conditions are negative predictors of improvement in renal function. In addition, in the subgroup of patients with hypercholesterolemia there was not significant reduction of glucose levels (Table 5). However, a low number of patients was present in this subgroup.

**Table 5:** Effect of hepatitis C virus clearance by direct‐acting antiviral agents at 24 weeks posttreatment on blood glucose, cholesterol and glomerular filtration rate (GFR) in the subgroups of the population with and without diabetes, hypercholesterolemia (≥ 200 mg/dL), hypertension, renal insufficiency (GFR ≤ 60 mL/min/1.73 m^2^), age (≥ 70 years) and the type of treatment used

Patients (*n* = 343)	Glycemia (mg/dL)	*P*	Cholesterolemia (mg/dL)	*P*	GFR (mL/min/1.73 m^2^)	*P*
Age ≥ 70 years (*n* = 145)						
Baseline	99.5 [89.8–117.8]	<0.014	152.5 [129.3–172.5]		81 [66–96]	
Posttreatment	95 [87–106.8]		168 [141.3–191.3]	<0.009	91 [72–102]	<0.004
Age < 70 years (*n* = 189)						
Baseline	99 [92–113]	<0.007	163 [135–185]		81.5 [76–109]	
Posttreatment	94 [88–106]		177 [151–203]	<0.001	92 [84–116.5]	<0.002
Diabetes (*n* = 74)						
Baseline	96 [86–105]		168 [148–187]		82 [72–102]	
Posttreatment	124.5 [118–140.5]	<0.001	190 [172–219]	<0.001	92 [78–112.5]	<0.007
No diabetes (*n* = 269)						
Baseline	103 [94–128]		154 [126.3–177.8]		81 [67.8–100]	
Posttreatment	90 [85–98]	<0.008	166 [138.3–192.8]	<0.016	91 [72–109]	<0.01
Hypercholesterolemia (*n* = 33)						
Baseline	103 [87–113]		211 [205–225]		84 [68–94]	
Posttreatment	99 [85–108]	<0.327	219.5 [211–227.3]	<0.179	87 [75–109]	<0.219
No hypercholesterolemia (*n* = 310)						
Baseline	102 [91.5–115]		153 [127–171]		81 [68.3–98]	
Posttreatment	94 [88–106]	<0.02	169 [142–191]	<0.001	92 [76–108]	<0.019
GFR ≤ 60 mL/min/1.73 m^2^ (*n* = 32)						
Baseline	106 [93–134]		133 [104.5–162]		54 [45–59]	
Posttreatment	98 [88–126]	<0.041	166 [127–183.3]	<0.001	58 [47–66]	<0.382
GFR > 60 mL/min/1.73 m^2^ (*n* = 311)						
Baseline	98 [90.5–113]		160 [133.8–182]		84 [74–102]	
Posttreatment	94 [88–104.8]	<0.087	174 [149–201]	<0.009	92 [78–109.8]	<0.014
Hypertension (*n* = 179)						
Baseline	99.5 [92–123.5]		155 [130.3–179]		78 [63–95.5]	
Posttreatment	96 [88–111.5]	<0.021	172 [149–201]	<0.004	84 [70–102]	<0.003
No hypertension (*n* = 164)						
Baseline	99 [90–112]		160 [135–182]		85.5 [76–105.5]	
Posttreatment	93 [87–104]	<0.032	176.5 [146.5–199]	<0.008	94 [80–112.3]	<0.001
SOF based treatment (*n* = 178)						
Baseline	103 [94–124]		154.5 [125–178.3]		81 [68–100.5]	
Posttreatment	96 [89–109.5]	<0.008	172.5 [143.5–200.3]	<0.001	90 [72–108]	<0.006
Non‐SOF‐based treatment (*n* = 165)						
Baseline	98 [89–110]		162 [136–182]		82 [69–98]	
Posttreatment	92 [85–104]	<0.019	174 [148.5–199]	<0.004	92 [77–108]	<0.003

SOF, sofosbuvir.

## Discussion

The results of our study show that patients who obtain HCV clearance by DAA treatments have significant changes in metabolism and renal parameters that must be known by physicians for proper post‐HCV clearance management.

HCV patients after viral clearance showed an overall improvement in glucose metabolism. A significant reduction in fasting blood glucose and insulin levels was observed and consequently a significant improvement in IR levels both in the group with mild to moderate fibrosis (F0–F2) and in those with advanced fibrosis (F3–F4). The data show that 89.6% of patients with IR before treatment had an improvement after HCV clearance, in 57.1% there was normalization of IR values and in 32.5% an improvement in IR levels. Furthermore, the data show an improvement in insulin sensitivity and a reduction of β‐cells pancreatic distress. Multivariate analysis shows that HCV clearance is an independent predictor of HOMA‐IR improvement (Table 3).

Furthermore, in patients with IFG, blood glucose levels normalized in 32.4% after SVR. Of great importance is also the fact that 44.6 of diabetic patients had an improvement in glycemic control and treatment with antidiabetic drugs was reduced or even suspended after the clearance of HCV. In agreement with our previous data,^12^ no significant change in glycemic parameters was observed in both untreated and non‐SVR patients. The data demonstrated that HCV clearance by DAAs is associated with significant changes in glucose metabolism in normoglycemia, IFG, and diabetic patients.

These data confirm our previous results obtained in HCV patients with advanced hepatic fibrosis (F3–F4) and without T2DM who demonstrated an improvement in blood glucose and IR levels, as well as an improvement in pancreatic β‐cell function and insulin sensitivity.^12^ Current data were obtained on a larger population, which also included patients with F0–F2 liver fibrosis and patients with IFG and T2DM. The data show that the improvement in glucose metabolism after HCV clearance is observed at all stages of hepatic fibrosis and severity of metabolic damage and that this improvement is independent of the BMI, since in these patients there was an increase in BMI during the observation period.

Other studies,^13^, ^14^, ^15^, ^16^, ^17^, ^18^, ^19^ except two,^20^, ^21^ confirm the positive impact of viral eradication obtained by DAAs on glycemic metabolism. They reported that SVR is accompanied by fasting blood glucose and HOMA reduction, improved glycemic control of diabetic patients and often by the need to reduce or stop hypoglycemic therapy.^13^, ^14^, ^15^, ^16^, ^18^, ^22^ We found that the negative predictive factors of an improvement were advanced hepatic fibrosis,^12^ other authors besides confirming the data showed that the family members for T2DM, a longer duration of diabetic disease and a high BMI also affect the nonimprovement of glucose metabolism.^16^, ^22^

Regarding the impact of viral clearance on the lipid profile, our data show a significant increase in LDL, and consequently in total cholesterol, irrespective of the degree of hepatic fibrosis. This increase is already present at the end of the treatment and tends to persist during the subsequent follow‐up. Although there is also an increasing trend of triglycerides and a reduction in HDL, these changes appear to be statistically significant only in the group with absent to moderate hepatic fibrosis. According to our previous data,^12^ no significant change in lipid parameters was observed both in untreated and non‐SVR patients.

Changes in lipid profile are reported in several studies^19^, ^21^, ^23^, ^24^, ^25^ which show that average levels LDL levels significantly increase significantly after viral eradication. The effect on triglycerides and HDL seems to be inconstant. Previously, obtaining SVR through IFN‐based regimens was also associated with changes in serum cholesterol levels.^26^, ^27^

The explanation for this phenomenon is probably to be found in the close link between HCV and the host's lipoproteins. The latter appear to be essential in all phases of the life cycle of the virus.^28^, ^29^ Chronic HCV infection is significantly associated with hypocholesterolemia (particularly with reduced LDL levels) compared with healthy controls, regardless of the degree of hepatic fibrosis.^27^, ^30^ Therefore, viral clearance is likely to restore basal lipid homeostasis, resulting in increased levels of LDL and total cholesterol.

In our study, patients who eliminated HCV by DAAs achieved improvement in renal function at the end of the observation period, regardless of the degree of hepatic fibrosis. In fact, our patients had an improvement of serum creatinine and an increase of eGFR of about 10% compared to the baseline value. Multivariate analysis shows that HCV clearance is an independent predictor of eGFR improvement (Table 4) and age a negative one.

As well documented in the literature, chronic HCV infection is associated with an increased risk of developing CKD.^5^, ^6^ The prevalence of HCV infection in hemodialysis patients is about 10 times higher than in the general population.^31^ CKD in presence of HCV infection appears to progress more rapidly toward ESRD^5^ and it is associated with a higher rate of death, acute coronary syndrome, arrhythmias, and transient ischemic attacks in comparison to CKD not associated with HCV infection.^32^ Chronic HCV infection can cause renal damage mainly by two mechanisms: a direct viral damage on renal parenchyma and an immune‐mediated mechanism.^33^

Few studies, mainly conducted in cryoglobulinemic patients, have investigated the impact of viral eradication achieved with DAAs on renal function showing an improvement in serum creatinine, proteinuria, and eGFR and a reduced risk of ESRD.^34^, ^35^, ^36^

Recently, the impact of DAA treatments on renal function has been evaluated in a retrospective study.^37^ The authors showed a significant improvement in eGFR in patients reaching SVR. Age and diabetes were negative predictors of improvement of renal function.^37^

The results of our prospective study confirm the data reported in the previous study^37^ showing that a significant improvement in renal function occurred after HCV clearance. However, contrary to what was reported in the previous work,^37^ our study shows that renal improvement occurred regardless of the presence of diabetes and the therapeutic regimen used. Our data show that a baseline eGFR ≤60 mL/min/1.73 m^2^ and an older age are negative predictor of improvement of renal function after HCV clearance by DAAs (Tables 4 and 5).

The reason of dissimilarities observed in the two studies on negative predictors of improvement in renal function are unclear, although some distinctions in the populations studied and in the approach to data analysis may have led to differences in results. The duration of diabetes in the two populations has not been evaluated and it is possible that a different duration of diabetes may have affected the results. A further factor which could have contributed to the disparity of the results on the factors associated with eGFR improvement could be the cause of the underlying renal damage in the two evaluated populations. Glomerular disease secondary to HCV infection (e.g. cryoglobulinemia) may improve after treatment with DAAs, while other causes may be less susceptible of improvement.

In conclusion, HCV clearance by DAA treatments induces a significant improvement in glucose metabolism and renal function, while causing an increase in the serum LDL and total cholesterol levels which require careful future monitoring. The data underline the importance of HCV eradication not only to prevent the progression of liver disease but also to improve or prevent HCV‐related metabolic and renal extrahepatic manifestations that play an important role in the morbidity and mortality of these patients.
